# SUMO control of centromere homeostasis

**DOI:** 10.3389/fcell.2023.1193192

**Published:** 2023-04-27

**Authors:** Sebastiaan J. W. van den Berg, Lars E. T. Jansen

**Affiliations:** ^1^ Department of Biochemistry, University of Oxford, Oxford, United Kingdom; ^2^ Instituto Gulbenkian de Ciencia, Oeiras, Portugal

**Keywords:** centromeres, CENP-A, epigenetics, SUMO, p97/Cdc48, sentrin/SUMO-specific protease

## Abstract

Centromeres are unique chromosomal loci that form the anchorage point for the mitotic spindle during mitosis and meiosis. Their position and function are specified by a unique chromatin domain featuring the histone H3 variant CENP-A. While typically formed on centromeric satellite arrays, CENP-A nucleosomes are maintained and assembled by a strong self-templated feedback mechanism that can propagate centromeres even at non-canonical sites. Central to the epigenetic chromatin-based transmission of centromeres is the stable inheritance of CENP-A nucleosomes. While long-lived at centromeres, CENP-A can turn over rapidly at non-centromeric sites and even erode from centromeres in non-dividing cells. Recently, SUMO modification of the centromere complex has come to the forefront as a mediator of centromere complex stability, including CENP-A chromatin. We review evidence from different models and discuss the emerging view that limited SUMOylation appears to play a constructive role in centromere complex formation, while polySUMOylation drives complex turnover. The deSUMOylase SENP6/Ulp2 and the proteins segregase p97/Cdc48 constitute the dominant opposing forces that balance CENP-A chromatin stability. This balance may be key to ensuring proper kinetochore strength at the centromere while preventing ectopic centromere formation.

## Introduction to the epigenetically heritable centromere

The ability to successfully partition replicated genomes to daughter cells during mitosis and meiosis is of vital importance to cells and organisms. Eukaryotic chromosomes feature centromeres, specialized genomic regions that assemble the kinetochore, a large multiprotein complex, during mitosis. These form the attachment site for spindle microtubules driving accurate genome segregation during cell division (McKinley and Cheeseman, 2016; Mellone and Fachinetti, 2021). Typically, centromeres assemble on long arrays of tandem repeats of AT-rich α-satellite DNA (Fukagawa and Earnshaw, 2014). These sequences nucleate both pericentric heterochromatin as well as the central kinetochore-forming domain that is defined by a unique chromatin structure featuring the histone H3 variant CENP-A (Ohzeki et al., 2002; Okada et al., 2007). Nucleation of a CENP-A domain is sufficient to trigger centromere formation, kinetochore assembly in mitosis, spindle microtubule binding and chromosome segregation (Barnhart et al., 2011; Mendiburo et al., 2011; Hori et al., 2013; Chen et al., 2015). Thus, CENP-A chromatin serves as the most upstream platform for centromere formation and is responsible for the nucleation of the constitutive centromere-associated network (CCAN) (Cheeseman and Desai, 2008) that in turn connects to the kinetochore complex (Foltz et al., 2006; Okada et al., 2006).

Moreover, once formed, CENP-A chromatin is heritable through a self-templating mode of CENP-A chromatin assembly (Jansen et al., 2007; Barnhart et al., 2011; Moree et al., 2011; McKinley and Cheeseman, 2016). While centromeric nucleosomes typically assemble on satellite DNA, this strong self–templated feedback loop can be maintained even on non-canonical DNA sequences. Both naturally occurring neocentromeres as well as experimentally induced ectopic centromeres (Marshall et al., 2008; Hori et al., 2013; Murillo-Pineda and Jansen, 2020; Murillo-Pineda et al., 2021) maintain CENP-A on non-satellite DNA. The ability of centromeres to form and be inherited in a manner largely uncoupled from DNA sequence elements serves as a paradigm of chromatin-based epigenetic memory. Understanding how CENP-A nucleosomes are replicated and passed on from one cell to the next is a central question in understanding their epigenetic transmission.

Consistent with a role in maintaining centromere identity, CENP-A nucleosomes are maintained with an unusually high stability in chromatin (Mitra et al., 2020b). Early fluorescence recovery after photobleaching (FRAP) and SNAP-tagging experiments in human cells revealed little turnover of centromeric CENP-A. Instead CENP-A nucleosomes are quantitatively transmitted through mitosis, diluted only by redistribution during DNA replication (Jansen et al., 2007; Hemmerich et al., 2008). CENP-A nucleosomes are more stable than other variants of H3, including H3.1 and H3.3 (Bodor et al., 2013). Interestingly, ectopically incorporated CENP-A turns over at rates similar to bulk chromatin, indicating that CENP-A is selectively stabilized at centromeres (Falk et al., 2015). One of the key factors that contributes to stabilizing CENP-A chromatin at centromeres is CENP-C that facilitates CENP-A nucleosomes compaction both *in vitro* and *in vivo* (Falk et al., 2015; Mitra et al., 2020a). Furthermore, the CENP-A chaperone HJURP, responsible for assembly of nascent CENP-A chromatin (Dunleavy et al., 2009; Foltz et al., 2009), also contributes to recycling centromeric CENP-A during DNA replication (Zasadzińska et al., 2018).

From this work a picture emerged that, once assembled, CENP-A chromatin is stably transmitted in a manner that is dependent on other centromere components. However, recent evidence has indicated that stability of CENP-A chromatin is context dependent. E.g., in mouse oocytes CENP-A appears to be remarkably stable with little turnover for up to a year in meiotically arrested cells (Smoak et al., 2016). On the other hand, in starfish eggs, CENP-A turnover appears much more prominent (Swartz et al., 2019). Further, in post-mitotic somatic cells, at longer timescales, CENP-A can gradually disappear from centromeres (Lee et al., 2010; Swartz et al., 2019). Estimates in *in vitro* senescent human somatic cells, revealed that while slow, turnover occurs at an estimated 10% per day (Swartz et al., 2019).

These recent findings indicate that CENP-A inheritance is regulated, even within the centromere complex, possibly depending on developmental cues. Recent discoveries revealed modification of CENP-A and the CCAN to be a potential means of regulation of CENP-A chromatin stability, including ubiquitylation (Hewawasam et al., 2010; 2014), phosphorylation (Bobkov et al., 2020). In this mini review we will discuss the emerging concept of SUMO regulation of centromeres and how it may play a central role in CENP-A stability and overall centromere homeostasis.

## The SUMO pathway

**FIGURE 1:**
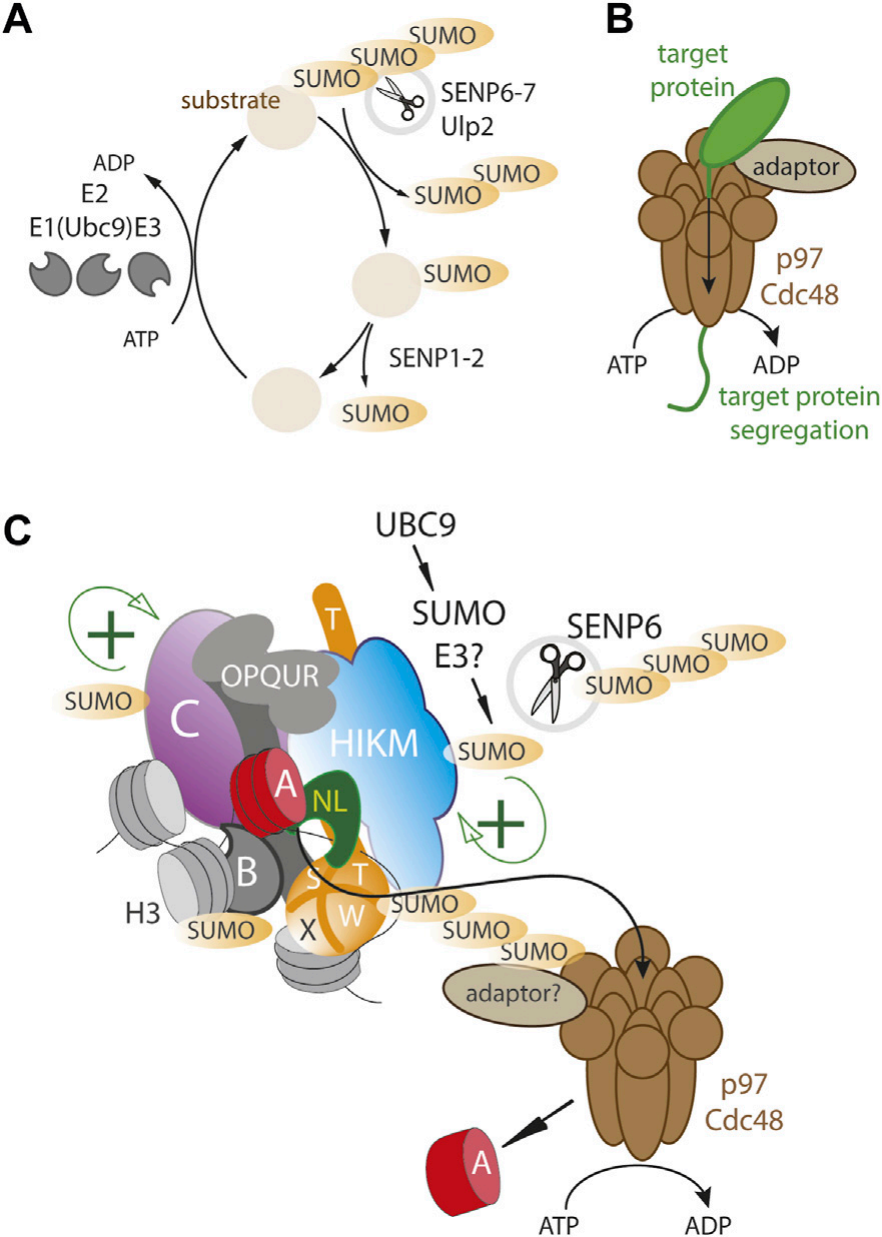
A dynamic SUMO balance maintains centromere homeostasis. **(A)** Basic outline of SUMO cycle where substrates are mono- and polySUMOylated by SUMO-specific E3 ligases in conjunction with the Ubc9 E2 ligase. SENPs deSUMOylate where SENP6-7/Ulp2 remove preferentially polySUMO chains **(B)** the VCP/p97/Cdc48 segregase is an ATP dependent motor protein complex that can physically remove proteins from protein assemblies and stable subcellular compartments **(C)** The human centromere complex (CCAN) is SUMOylated at multiple sites (including CENP-C, -B, -T, -H, -I -K), possibly facilitating complex formation and stability (green+). Excessive SUMOylation results in a p97/Cdc48 dependent removal of the CCAN as well as CENP-A. SENP6/Ulp2 counteracts p97/Cdc48 by continuously removing polySUMO chains.

The Small Ubiquitin like Modifier (SUMO) was discovered as a ubiquitin-like protein (Boddy et al., 1996; Matunis et al., 1996; Okura et al., 1996; Mahajan et al., 1997), that is attached to a substrate by E1, E2 and E3 enzymes in a similar fashion as ubiquitin (Gong et al., 1997; Johnson and Gupta, 2001; Kahyo et al., 2001; Pichler et al., 2002). In humans, three different functional SUMO isoforms (SUMO1-3) exist (Gareau and Lima, 2010; Flotho and Melchior, 2013). While SUMO1 is distinct, SUMO2 and SUMO3 are highly similar and are considered functionally equivalent (Flotho and Melchior, 2013). The E1 enzyme is a hetero dimer of SAE1 and SAE2, activates the SUMO protein and transfers it to the only known E2 Ligase UBC9 (Figure 1A) (Desterro et al., 1997; Gong et al., 1997). The E2 ligase, together with an E3 ligase (e.g., the family of PIAS proteins PIAS1-4) transfers the SUMO to a lysine residue on the substrate via an isopeptide bond (Kahyo et al., 2001; Pichler et al., 2002). Similar to ubiquitin, SUMO can be elongated into poly-SUMO chains predominantly through chain formation on lys11 on SUMO2/3, but alternative lysines can be used leading to branching of the poly-SUMO chain (Tatham et al., 2001; Matic et al., 2008). PolySUMOylation is highly reversible through the action of deconjugating enzymes named Sentrin/SUMO-specific proteases (SENPs) that are able to remove the SUMO from the substrates (Gareau and Lima, 2010; Nayak and Müller, 2014; Jansen and Vertegaal, 2021). Typically, complexes of substrates are modified at multiple residues and SUMO appears to act as a platform for multivalent protein-protein interactions helping to stabilize protein assemblies (Hay, 2005). Furthermore, through the action of SUMO-dependent ubiquitin ligases (STUbLs), polySUMO chains can be polyubiquitylated. In this way SUMOylation can serve not only to stabilize proteins complexes but also as a trigger for controlling protein turnover (Prudden et al., 2007; Perry et al., 2008).

## SUMO regulation of the centromere

The link between SUMO and centromere biology is as old as the SUMO field itself, where SMT3, the sole SUMO homolog in budding yeast, was originally isolated as a high-copy suppressor of mutations in MIF2, the budding yeast homolog of CENP-C (Meluh and Koshland, 1995). This genetic interaction is also observed for a temperature sensitive allele in vertebrate chicken cells (Fukagawa et al., 2001), suggesting a conserved mode of regulation. In the yeast *Saccharomyces cerevisiae*, several centromere proteins are SUMOylated, including Ame1^CENP-U^, Okp1^CENP-Q^, Mcm16^CENP-H^ and Mcm22^CENP-K^ and Mcm21^CENP-O^, which is functionally required for high fidelity chromosome segregation (Suhandynata et al., 2019). SUMO modification of these proteins is kept to a low level by the key SUMO protease Ulp2 that is targeted to the centromere via direct interaction with Ctf3^CENP-I^. This is critical for maintaining low SUMOylation levels and preventing mitotic errors (Suhandynata et al., 2019; Quan et al., 2021).

Similar to yeast, in human cells the CCAN proteins CENP-I, CENP-C, CENP-H and CENP-T are also found to be SUMOylated (Mukhopadhyay et al., 2010; Liebelt et al., 2019; Mitra et al., 2020a). Depletion of the Ulp2 homolog SENP6 that selectively targets poly-SUMO2 and SUMO3 chains (Mukhopadhyay and Dasso, 2007), results in excessive polySUMOylation of CCAN proteins, leading to their delocalization from the centromere (Mukhopadhyay et al., 2010; Liebelt et al., 2019; Mitra et al., 2020a). These findings suggest that low level SUMOylation of CCAN proteins may be functionally important but that excessive SUMOylation can result in the disassembly of the centromere complex which is kept in check by deSUMOylases (Figure 1). This creates an opportunity for controlling the size and stoichiometry of the centromere complex. While yeast Ulp2 is targeted to centromere thereby locally stabilizing centromere proteins, there is, as of yet, no direct evidence of SENP6 targeting to CCAN proteins in humans, nor is SENP6 enriched at the centromere as assessed by imaging (Liebelt et al., 2019; Mitra et al., 2020a).

## The role of SUMO in the control of CENP-A assembly and maintenance

Interestingly, changes in the SUMO balance by SENP6 depletion, not only affect CCAN levels but also dramatically affect human CENP-A chromatin stability (Liebelt et al., 2019; Mitra et al., 2020a), indicating that CENP-A nucleosomes, while stable at centromeres, can be rapidly turned over in a SUMO-dependent manner. However, in contrast to the CCAN components mentioned above, CENP-A itself does not appear to be SUMOylated indicating its levels are controlled via SUMOylation of the downstream CCAN components (Liebelt et al., 2019; Mitra et al., 2020a). The dynamics of centromere protein loss upon SENP6 depletion showed that while the CCAN proteins CENP-T, CENP-I, CENP-H and CENP-C are rapidly delocalized from the centromere, CENP-A is removed with a delay, indicating its loss is secondary to CCAN protein removal (van den Berg et al., 2023) (Figure 1C).

Nucleosomes are highly stable complexes, maintained by numerous multivalent interactions between histones and DNA (Andrews and Luger, 2011). Therefore, disruption and turnover of CENP-A *in vivo* likely requires an energy dependent mechanism. Previously identified disruptive forces that can drive CENP-A turnover are transcription (Hill and Bloom, 1987; Nakano et al., 2008), replication (Nechemia-Arbely et al., 2019) as well as chromatin remodeling activity (Perpelescu et al., 2009).

Recent inquiries into the role of SUMO in CENP-A turnover led to another possible mechanism of energy dependent centromere disruption, the AAA+ ATP-dependent segregase p97 also known as valosin-containing protein (VCP) or Cdc48 in worms and yeast (Torrecilla et al., 2017). p97 consists of a homohexameric barrel-like structure with two ATPase domains (DeLaBarre and Brunger, 2003; Huyton et al., 2003) that is able to tread a polypeptide thereby disrupting stable protein assemblies (Figure 1B). p97 targets a wide range of clients through a large number of adaptor proteins (Schuberth and Buchberger, 2008; Yeung et al., 2008; Meyer et al., 2012). Classically, p97 targets are ubiquitylated and p97 adaptors often carry ubiquitin binding motifs (Yeung et al., 2008). Many clients are cytoplasmic but recent evidence has also identified nuclear targets, e.g., Aurora B is removed by p97 during mitosis, required to maintaining mitotic fidelity (Ramadan et al., 2007; Dobrynin et al., 2011). While most commonly targeted via ubiquitin signals, p97 has also been shown to be targeted via SUMO binding adapters (Yeung et al., 2008; Bergink et al., 2013; Gibbs-Seymour et al., 2015).

Recent work revealed that the SUMO-dependent turnover of the human CCAN is dependent on p97 (van den Berg et al., 2023). Interestingly, while CENP-A itself does not appear to be SUMOylated (Liebelt et al., 2019; Mitra et al., 2020a), it binds to p97 and its turnover is mediated by p97 in a SUMO-dependent manner, indicating that p97 is recruited to extract CENP-A via other CCAN members. These findings indicate a SUMO-dependent p97/SENP6 regulatory axis that either stabilizes or destabilizes CENP-A chromatin (Figure 1C). This may be important to dynamically maintain accurate homeostasis of the centromere complex, thereby regulating kinetochore strength that may be important for a balanced mitotic spindle (Drpic et al., 2018). Moreover, p97 has been shown to control centromeric CENP-A levels in a developmental context. In *Arabidopsis thaliana*, p97 removes CENP-A in a SUMO-dependent manner selectively in non-dividing pollen vegetative cells during pollen tube formation (Mérai et al., 2014). In this case CENP-A itself is SUMOylated and p97 targets CENP-A through its binding partners Npl4 and Ufd1. p97 action leas to centromere loss and chromatin decondensation which is critical for pollen tube development. How p97 is targeted to CENP-A remains to be answered. A recent attempt to identify adaptors found no evidence for the involvement of the canonical factors NPL4 and UFD1, suggesting p97 is targeted by more specialized adaptors (van den Berg et al., 2023).

## Is there a role for SUMO-dependent ubiquitylation of centromere proteins?

PolySUMOylation can become a substrate for polyubiquitylation via the action of SUMO-targeted E3 ubiquitin ligases (STUbLs). To what extend this occurs at centromeres is still unclear. Early work on CENP-I showed that its SUMOylation results in its subsequent degradation and depletion of the STUbL RNF4 stabilized polySUMOylated CENP-I. This suggests RNF4 ubiquitinates polySUMOylated CENP-I (Mukhopadhyay et al., 2010), although this was not directly tested. As outlined above, in the absence of SENP6, most CCAN proteins as well as CENP-A are lost from centromeres (Fu et al., 2019; Liebelt et al., 2019; Mitra et al., 2020a). There has been some evidence that RNF4 is involved, as depletion of RNF4 can rescue centromeric CENP-A levels that are lost in a SENP6 mutant (Fu et al., 2019). However, a recent study could not corroborate this observation (van den Berg et al., 2023). Instead, depletion of RNF4 resulted in a loss of CENP-A rather than suppress excessive SUMOylation, suggesting that RNF4 may play a more direct positive role in maintaining CENP-A. However, dissecting the contribution of RNF4 is complicated by the finding that the SUMO E2 and E3 ligases that generate SUMO chains are themselves a target for RNF4-mediated ubiquitylation, creating a feedback mechanism where RNF4 controls the levels of SUMOylation (Kumar et al., 2017). This may in part explain why loss of RNF4 can, indirectly, suppress a SENP6 defect.

Further, direct analysis of CENP-K and CENP-T showed these CCAN proteins to be ubiquitylated only at low levels. Proteasome inhibition did not stabilize these proteins, indicating these SUMOylated CCAN proteins are not targeted for a STUbL-dependent degradation (Liebelt et al., 2019). Additionally, RNF4 depletion did not stabilize SUMOylated CCAN proteins. Both CENP-C and CENP-A do not appear to be degraded upon SUMOylaiton (Mitra et al., 2020a), indicating the SUMO2/3 signal on the CCAN has a non-canonical function. Thus while CENP-I may be turned over by proteolysis (Mukhopadhyay et al., 2010) this appears not to be a general theme for the CCAN.

Interestingly, the key CENP-A assembly factor Mis18BP1 has also been reported to be SUMOylated in a manner that is under SENP6 control (Fu et al., 2019). In the absence of SENP6, Mis18BP1 becomes hyperSUMOylated in a PIAS4 E3 ligase-dependent manner. In this case, SUMOylation results in subsequent targeting by RNF4 for polyubiquitylation ubiquitylation and targeting for proteasomal degradation (Fu et al., 2019; Liebelt et al., 2019). This results in the loss of a new assembly of centromeric CENP-A and causes a disruption in the self-templated epigenetic feedback loop of CENP-A. It is not clear what the role is of Mis18BP1 SUMOylation under physiological conditions and whether SUMOylation also plays a positive role in the CENP-A assembly process.

## The role of SUMO-mediated ubiquitylation in removal of ectopic CENP-A

**FIGURE 2:**
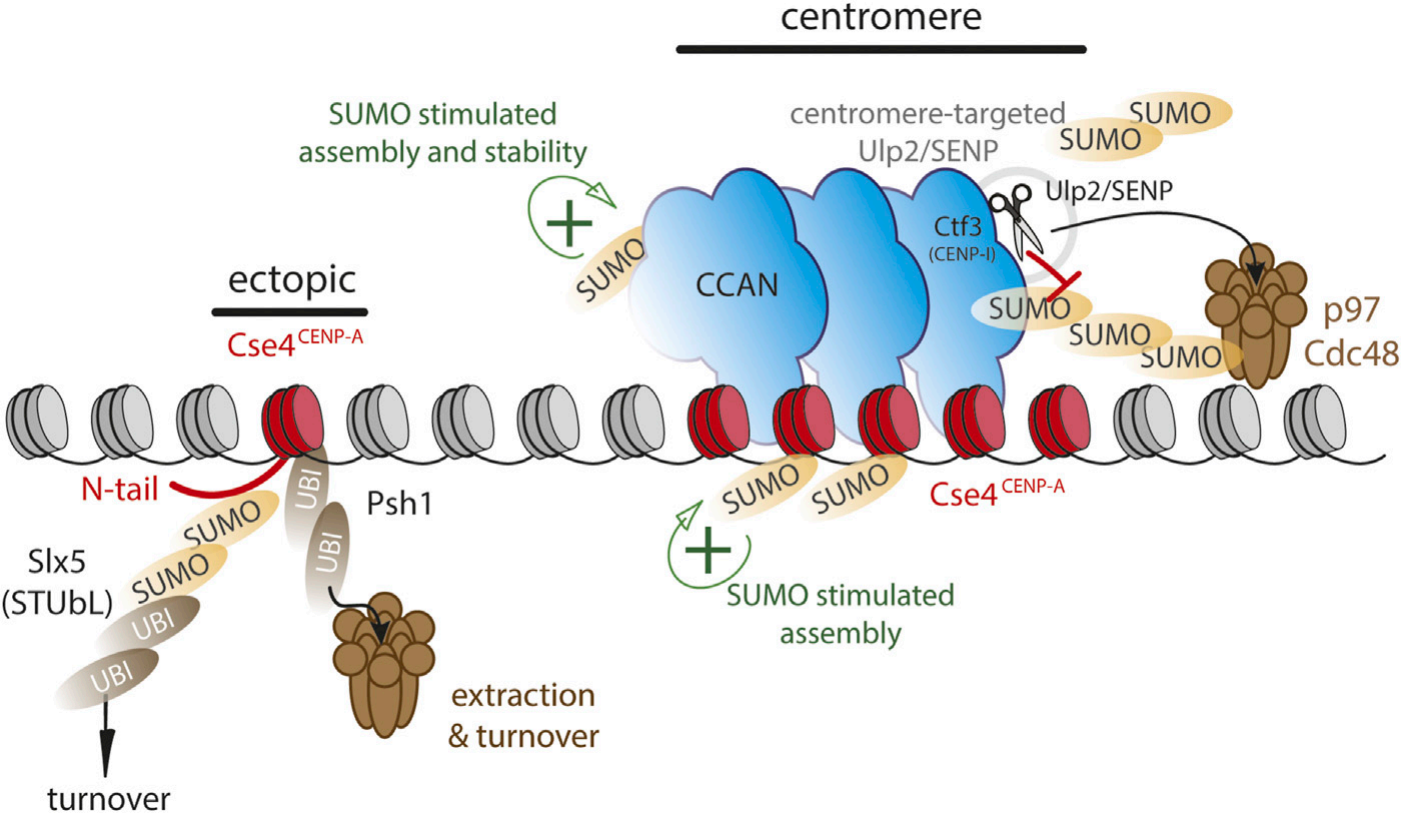
SUMO and ubiquitin dynamics may differentially control CENP-A chromatin stability at centromeres versus non-centromeric loci. Model largely based on evidence from budding yeast. Functional SUMO at centromeres (green+) is kept at a low level by targeting Ulp2/SENP to centromeres via CENP-I/Ctf3, thereby stabilizing the centromere complex. Assembly of CENP-A/Cse4 is stimulated by C-terminal SUMO. Ectopic loci are vulnerable to polySUMOylation as well as SUMO-dependent ubiquitylation via Slx5 on Cse4 N-terminus or directly via Psh1 ubiquitin ligase, targeting CENP-A/Cse4 to Cdc48/p97 segregase for chromatin extraction.

In the yeast *S. cerevisiae,* the deposition of non-centromeric CENP-A (Cse4) is facilitated by SUMOylation near its C-terminus, suggesting a positive role for SUMO in centromere assembly (Ohkuni et al., 2020). However, SUMOylation can also drive turnover. SUMO modification of the Cse4 N-terminus by the SUMO E3 ligases Siz1 and 2 renders it a substrate for the STUbL Slx5 that in turn ubiquitinates SUMOylated Cse4 and targets it for degradation (Ohkuni et al., 2016; 2018). The dual SUMOylation of CENP-A highlights its differential roles as the N-terminal SUMOylation leads to decreased non-centromeric assembly while C-terminal SUMOylation promotes the non-centromeric assembly (Figure 2). Mislocalized CENP-A can also become a substrate for the E3 ubiquitin ligase Psh1 for polyubiquitination (Hewawasam et al., 2010; 2014; Ranjitkar et al., 2010). Psh1-mediated ubiquitylation does not appears to be SUMO-dependent and in this case Cse4 modification renders it a target for the p97 homolog Cdc48 thereby removing it from the chromatin, revealing a potential analogous mechanism in yeast and humans, although through different signals (Ohkuni et al., 2022; van den Berg et al., 2023) (Figure 2). In human cells, non-centromeric CENP-A has been shown to turn over more rapidly than the centromeric pool (Falk et al., 2015). Moreover, while SUMO-driven turnover can occur throughout the cell cycle (Mitra et al., 2020a), non-centromeric CENP-A appears to be turned over largely during S phase (Nechemia-Arbely et al., 2019). It will be interesting to determine whether the SUMO/p97 mechanism plays a role in CENP-A removal at this stage.

## Discussion: emerging roles of SUMO in controlling centromere homeostasis

The current literature on SUMO control of centromere maintenance identifies a destructive role where polySUMOylation is primarily driving disassembly and turnover via p97, countered by SENP6. A key point that is merging is that CENP-A maintenance by these opposing forces occurs not only to remove CENP-A from ectopic sites as shown in yeast (Ohkuni et al., 2022) but even at the centromere itself (Mitra et al., 2020a; 2020b). This dynamic balance is maintained throughout the cell cycle (Mitra et al., 2020a; van den Berg et al., 2023) and low level p97-dependent turnover occurs even under conditions where SENP6 is functional and SUMOylation levels of the CCAN are low (van den Berg et al., 2023). This suggests that continuous SUMOylation of the CCAN components allows for a dynamic regulation of centromeric levels. While a direct physiological role of SUMO/p97 remains to be tested, it may be involved in maintaining a balanced centromere strength in mitotic and meiotic cells and be subject to development cues such as CENP-A loss in non-dividing cells (Lee et al., 2010; Swartz et al., 2019) or reducing centromere size in stem cells (Milagre et al., 2020), analogous to its role in plant development (Mérai et al., 2014).

Importantly, underlying the seemingly destructive force of polySUMOylation, there may be a functional requirement for SUMO moieties, where limited SUMOylation may have a role in stabilizing interactions within the centromere complex as has been shown in yeast (Suhandynata et al., 2019; Quan et al., 2021). This makes SUMO a double-edged sword where limited SUMOylation (e.g., monoSUMO) is required for centromere function. Indeed, depletion of the SUMO E3 ligase PIAS4 results in reduction of CENP-A levels (van den Berg et al., 2023), suggesting that some SUMOylation is beneficial for centromere maintenance. Furthermore, it is noteworthy that SENP6, the key deSUMOylase involved in human centromere maintenance is able to deconjugate a polySUMO chain, but is reportedly very inefficient in removing the final SUMO moiety from its substrate (Jansen and Vertegaal, 2021). This suggest that at steady state, the PIAS/SENP6 balance results in net mono-SUMOylaiton of centromere proteins. Identifying the functional role of SUMOylation at the centromere and discovering to what extend is it necessary for centromere function and stability, is a key question going forward. In other assemblies SUMO has been known to act as a molecular ‘glue’ e.g., in PML bodies, allowing them to form a more stable complex through numerous multivalent interactions between SUMO and SUMO interacting proteins (Geoffroy et al., 2010; Erker et al., 2013; Sahin et al., 2014; Banani et al., 2016). Potentially, a similar role can be envisaged for the SUMOylation of the CCAN.

The putative functional requirement for limited SUMOylation while excessive SUMOylation is detrimental requires an optimal level and thus tight regulation of the SUMOylation events at the centromere. This allows for a rapidly adaptable CCAN that can either shrink or grow during stochastic fluctuations and disruptive events such as transcription, DNA damage or even pulling forces by the mitotic spindle during mitosis. The central outstanding questions are; what is the functional role of SUMO, what are its relevant CCAN targets and does SUMO-driven turnover serve as a mechanism to selectively stabilize CENP-A at the centromere, while preventing ectopic accumulation elsewhere?
